# Do Radicalized Minors Have Different Social and Psychological Profiles From Radicalized Adults?

**DOI:** 10.3389/fpsyt.2019.00644

**Published:** 2019-09-10

**Authors:** Alice Oppetit, Nicolas Campelo, Laura Bouzar, Hugues Pellerin, Serge Hefez, Guillaume Bronsard, Dounia Bouzar, David Cohen

**Affiliations:** ^1^Service de Psychiatrie de l’Enfant et de l’Adolescent, Hôpital Pitié-Salpêtrière, Paris, France; ^2^ARTEMIS, Atelier de Recherche, Traitement et Médiation Interculturelle et Sociale, Paris, France; ^3^Laboratoire PCPP- EA 4056, Institut de Psychologie, Université Paris V René Descartes SPC, Paris, France; ^4^CPDSI and Bouzar-Expertises, Paris, France; ^5^CMPPD, Conseil Départemental des Bouches-du-Rhône, Marseille, France; ^6^Laboratoire de Santé Publique, EA3279, Aix Marseille Université, Marseille, France; ^7^Publlic Health Department, EA 7479, SPURBO, Brest, France; ^8^Institut des Systèmes Intelligents et de Robotiques, Université Pierre et Marie Curie, Paris, France

**Keywords:** radicalization, terrorism, violent extremism, social context, adolescence

## Abstract

**Introduction:** Radicalization is a major issue in Western societies. Supposedly, there is no predefined pathway leading to radicalization. However, youth appears to be at risk for radicalization. The aim of this study was to compare the social and psychological profiles of radicalized minors and radicalized adults.

**Methods:** This cross-sectional study is based on the first large prospective sample of young French individuals (N = 150) who aimed to join the Islamic State (IS) between 2014 and 2016. This sample included 70 adolescents (mean age 15.82 years old, SD 1.14) and 80 young adults (mean age 23.32 years, SD 4.99). We compared the two groups on their sociodemographic and psychological characteristics.

**Results:** Radicalized minors and radicalized adults have different profiles and follow different paths in the radicalization process. Among the group of minors, there are significantly more female subjects (81.4% versus 55.0%, adj. p = 0.007) and more self-harm history before radicalization (44.3% versus 16.2%, p <0.001). In addition, there are significantly less attempts to radicalize the entourage (24.3% versus 50.0%, adj. p = 0.007), and a tendency to show less cases of radicalization among the entourage (32.9% versus 52.5%, adj. p = 0.075) and less radicalization through physical encounter (45.7% versus 65%, adj. p = 0.082).

**Discussion:** Overall, radicalized minors appear to be more psychologically vulnerable individuals than radicalized adults. These differences highlight the importance of tailored interventions in order to prevent radicalization among vulnerable adolescents.

## Introduction

Terrorism is a persistent global threat for security, for international stability and prosperity (1). According to the Global Attack Index, 22,487 terrorist attacks have been committed worldwide in 2017, the Islamic State being the most active group (2). Although the Islamic State has been defeated recently in Iraq and Syria, its leader Abou Bakr Al-Baghdadi sent an audio message in August 2018 encouraging terrorists to continue Jihad in the Western World. Fighting terrorism is a top priority for the international community: since 1963, nineteen international legal instruments have been developed under the auspices of the United Nations in order to prevent terrorism. The understanding of terrorist acts has changed in recent years in Europe with the increase of ‘homegrown’ terrorists, born and raised in Europe, who adopt the ideology of violent radical Islamism (3–5). In France, the Government listed 1,704 French people in May 2015 who either joined the IS in Syria, returned from Syria, were on their way to Syria or said they want to join the IS, with a 300% increase between January 2014 and May 2015 (6). Beyond the repressive and security apparatus, many researchers and professionals from the fields of anthropology, political science, sociology, psychology, and psychiatry have been involved in understanding radicalization in order to prevent terrorism.

Researches from different fields have confirmed that there is no predefined pathway leading to radicalization: radicalized individuals come from various backgrounds, have different origins, different family beliefs, social status or gender (4, 7–9). Many studies from sociology and political science argue that it is not possible to adopt a sociological determinist vision of terrorist acts (Hamas, IRA, ETA, Al-Qaida, etc.). Social factors such as educational level, unemployment, and occupational integration have some weight but are insufficient to explain radicalization phenomenon as all these factors depend on the cultural contexts (5, 10–12). The multiplicity of factors (individual trajectory, group belonging, and social context) prevents the comprehension of radicalization as a single pathway. Also, the recruitment of organization members and the method of violent actions are very different between Al-Qaida and ISIS (13). Besides, some of the radicalized individuals come from marginalized communities, but it is not the case for all (14). These considerations emphasize the importance of considering social, cultural and political context in the radicalization phenomenon. However, youth appears to be a risk factor for radicalization: since 2010, radicalized individuals in Europe are younger than they used to be (often teenagers) and the number of young women involved is increasing (5). In France, 1,017 minors were registered in 2015 with the French judiciary authorities by the police because of objective and worrying signs evoking a radicalization process (6). Existing studies on radicalization have mainly focused on adults (15–20) and have scarcely explored specifically the question of radicalized adolescents (21–24).

However, to fight against radicalization at best, it is important to approach the reality of the field as closely as possible and to distinguish the different types of radicalized individuals. In order to do so, we analyzed the data from the first large prospective sample of French individuals (mean age: 19.82 years) who aimed to join the IS between 2014 and 2016 and were followed-up for 2 years in average (25). At FU, 23% were still radicalized or had reached the Islamic State. Multivariate models showed that worse status at FU was predicted by being married, having married parents, having attempted to radicalize other relatives, and having a close friend or relative imprisoned before radicalization (20). The fact that adolescence is a phase of psychological turbulence and reorganization suggests that the motives for radicalization may differ between a 13-year-old minor and a 30-year-old adult. In addition, society has a mission of child protection for minors and must do its best to protect these vulnerable citizens. The objective of the current study is to decipher the profiles of French minors who wanted to join the IS between 2014 and 2016 and compare the group of minors versus the group of adults on different variables of interest.

## Methods

### Design of the Study

This observational cross sectional study is based on the first large sample of young French individuals who aimed to join the IS between 2014 and 2016. This study is ancillary to a prospective study whose objective was to explore the motivations of radicalized individuals and the characteristics that predicted prognosis in terms of de-radicalization at 2 year-follow-up (25). All individuals who contacted the CPDSI between January 2014 and December 2015 were included. The original prospective study mixed qualitative and quantitative approaches. Based on case-by-case interactions with CPDSI professionals who were responsible for monitoring these individuals, the team collected information by studying computer visited websites and mails, by gathering information from police investigation when available, and by conducting (i) semi-structured individual interviews with the young people and/or their families or (ii) group therapy sessions. Individuals for whom more than 5% of the data was missing were excluded. These data enabled the CPDSI team to perform a qualitative analysis and to distinguish 8 motivational profiles that they named after mythologic or metaphoric references. Besides, the data included some quantitative information such as sociodemographic, individual and family characteristics, police intervention, personal history before radicalization, family history, police history before radicalization. In the present observational study, we focused on the quantitative data in order to compare at baseline the profiles of radicalized minors versus radicalized adults. Variables available in the dataset are listed in **Table 1**.

**Table 1:** Characteristics of the sample and comparison between the group of radicalized minors and radicalized adults.

	Total (N = 150)	Minors (N = 70)	Adults (N = 80)	p	Adjusted p
Socio-demographic characteristics					
Age in years, mean (SD) [range]	19.82 (5.28) [13–40]	15.82 (1.14) [13–17]	23.32 (4.99) [18–40]	**<0.001**	**<0.001**
Gender (Female)	67.3%	81.4%	55%	**<0.001**	**<0.007**
Family status (Single)	72.7%	88.6%	58.8%	**<0.001**	**<0.001**
Children (Yes)	21.3%	4.3%	36.2%	**<0.001**	**<0.001**
Parents’ marital status (Married)	44%	48.6%	40%	0.291	0.552
Atheist (Yes)	39%	40.0%	38.8%	0.876	0.977
Muslim cultural background (Yes)	37.3%	38.6%	36.2%	0.769	0.9
Christian cultural background (Yes)	32.0%	35.7%	28.7%	0.362	0.642
Other cultural background (Yes)	6.67%	12.9%	1.2%	**0.006**	**0.037**
Personal history					
Fusional relationship before radicalization (Yes)	60.0%	58.6%	61.3%	0.738	0.897
Relationship of influence before radicalization (Yes)	46.0%	45.7%	46.2%	0.948	1
Self-harm history before radicalization (Yes)	29.3%	44.3%	16.2%	<0.001	<0.001
Hospitalization in psychiatric ward before radicalization (Yes)	12.7%	12.9%	12.5%	0.948	1
Psychiatric follow-up before radicalization (Yes)	35.3%	41.4%	30%	0.144	0.356
Personal physical health issue before radicalization (Yes)	18.7%	20%	17.5%	0.695	0.882
Depression of the subject before radicalization (Yes)	44.0%	41.4%	46.2%	0.553	0.814
Physical or sexual abuse of the subject (Yes)	26.7%	30%	23.8%	0.388	0.647
Neglect or psychological abuse of the subject (Yes)	85.3%	87.1%	83.8%	0.558	0.814
Experience of abandonment before radicalization (Yes)	82.0%	77.1%	86.2%	0.148	0.356
Personal addiction and drug abuse (Yes)	22.0%	17.1%	26.2%	0.179	0.379
Imprisonment before radicalization (Yes)	16.0%	11.4%	20%	0.153	0.356
Educational follow-up before radicalization (Yes)	22.0%	27.1%	17.5%	0.155	0.356
Family history					
Addiction and drug abuse of a relative (Yes)	32.0%	31.4%	32.5%	0.888	0.977
Rape or abuse of a relative (Yes)	16%	14.3%	17.5%	0.592	0.814
Physical abuse of a relative (Yes)	32.0%	34.3%	30%	0.575	0.814
Depression of a relative before radicalization (Yes)	40.7%	41.4%	40%	0.859	0.977
Physical health issue of a relative (Yes)	27.3%	28.6%	26.2%	0.75	0.897
Radicalization process and what happens after radicalization					
Previous cases of radicalization among the entourage (Yes)	43.4%	32.9%	52.5%	**0.015**	**0.075**
Attempts to radicalize the entourage (Yes)	38.0%	24.3%	50%	**0.001**	**0.007**
Radicalization via internet (Yes)	99.3%	100%	98.8%	1	1
Radicalization via physical encounter (Yes)	56.0%	45.7%	65%	**0.018**	0.082
Educational follow-up after radicalization (Yes)	43.0%	58.6%	28.7%	**<0.001**	**<0.001**
Still considered as Muslim after deradicalization (Yes)	92.0%	85.7%	97.5%	**0.008**	**0.044**
Motivational dimensions for radicalization: factorial classification*					
Factor 1: Violence and megalomania	0.82(1.26)	0.86(1.21)	0.78(1.33)	0.503	NA
Factor 2: Depression and abuse	0.82(1.05)	0.94(1.23)	0.69(0.8)	0.363	NA
Factor 3: Responsibility and guilt	0.82(1.11)	0.83(1.31)	0.81(0.86)	0.357	NA
Factor 4: Loneliness and poor insight	0.82(1.06)	0.85(1)	0.79(1.14)	0.329	NA
Factor 5: Responsibility and sacrifice	0.82(1.56)	0.94(2.03)	0.69(0.76)	0.422	NA
Factor 6: Violence and uncertainty	0.82(1.08)	0.68(0.77)	0.97(1.34)	0.434	NA
Factor 7: Issue with sexuality	0.82(1.5)	0.9(1.62)	0.73(1.36)	0.78	NA
Factor 8: Loneliness and sensitivity	0.82(1.07)	0.86(1.18)	0.77(0.96)	0.904	NA
Motivational dimensions for radicalization: qualitative classification**					
Fortress	4.7%	5.7%	3.8%	0.706	0.882
Zeus	10%	11.4%	8.8%	0.585	0.814
Suicidal	15.3%	21.4%	10%	0.053	0.208
Lancelot	18.7%	15.7%	21.2%	0.385	0.647
Savior	14.7%	10%	18.8%	0.131	0.356
IS as Utopia	33.3%	31.4%	35%	0.643	0.852
Mother Teresa	20.7%	25.7%	16.2%	0.153	0.356
Sleeping Beauty	24%	37.1%	12.5%	**<0.001**	**<0.001**

*Details on the factorial analysis are available in Campelo et al. (25); **Details on the qualitative classification are available in Bouzar et Martin (21) and Bouzar (26). In the case of the qualitative dimension, each individual only had one dimension attributed leading to a percentage. Bolded texts indicate the difference is significant between the two groups i.e. p < 0.05.

### Participants

A total of 150 individuals were included (mean age: 19.82 years (±5.28) [range:13–40]; 101 (67.3%) females; 100 (67%) convert Muslims). The characteristics of the sample are summarized in **Table 1** (left column). The sample included 101 females and 49 males. Mean age was 19.82 (±5.28) years. To assess the representability of the sample, we compared the sociodemographic characteristics of our sample with that of all individuals registered for radicalization from the French Home Office during the same period. In comparison, we included individuals mainly from the Paris area and South-Eastern France. Additionally, our sample included significantly more females, more individuals who were convert Muslims, and more minors. However, the rate of individuals who reached IS (10%) was similar to that of the national estimate (6, 25).

### Statistical Analysis

The aim of this study was to compare the profiles of radicalized minors versus radicalized adults in order to determine the specificities of radicalized minors. Among the 150 individuals included in the study, 70 were minors (age <18 years old), and 80 were adults (age ≥18 years old). In order to compare the two groups, we performed chi-2 analysis for the different nominal qualitative variables of interest when no expected count under the null hypothesis was less than 5 (otherwise, Fisher exact test was performed). For quantitative variables, we used t-test in cases of normal distribution and homogeneous variance in each group (and Wilcoxon rank sum test, otherwise). To take multiple comparisons into account despite the number of subjects included, we calculated adjusted p using false discovery rate (FDR) for all comparisons. We chose a FDR at 5%, meaning that we only accepted 5% of false positive (21). In **Table 1**, we indicate both p and adjusted p (adj. p).

## Results

The results of the statistical analysis are presented in **Table 1**. In the group of minors, the mean age was 15.82 years old (SD 1.14). In the group of adults, the mean age was 23.32 years old (SD 4.99). Univariate analyses reveal significant differences between adolescents and young adults that refer to differences in sociodemographics, in psychopathological profiles and in the radicalization process.

First, there are some important social and psychological differences between the two groups. In the group of radicalized minors, there were significantly more female subjects than in the group of adults (81.4% versus 55.0%, adj. p = 0.007). Moreover, we observed that the minors had more self-harm history before radicalization (44.3% for minors versus 16.2% for adults, adj. p < 0.001) and that, after having come out of the process of radicalization, there were fewer minors who were still considered as Muslims than in the group of de-radicalized adults (85.7% versus 97.5%, adj. p = 0.044), suggesting that the identifications of adolescents are more labile than those of adults.

Second, the table highlights some significant differences in the conditions of the radicalization process. Although radicalization *via* internet is omnipresent among the two groups (149 out of 150 subjects), we found that less adolescents attempted to radicalize their entourage (24.3% adolescents versus 50.0% adults, adj. p = 0.007. The ‘entourage’ was defined as close friends and family. Also, there was a tendency to have less cases of radicalization among the entourage in the group of minors (32.9% versus 52.5%, adj. p = 0.075 and p = 0.015); and less radicalization through physical encounter (45.7% versus 65%, adj. p = 0.088 and p = 0.018) These findings suggest that for adults, the radicalization occurs also by networks of *proximity* (neighborhood, entourage, physical contact) whereas for minors it takes place more exclusively by the Internet and virtual contacts.

Third, in terms of motivational dimensions and profiles, we found no significant difference between minors and adults, except for the ‘Sleeping Beauty’ profile (22) that was significantly more frequent in minors (**Table 1**). This profile was defined during the qualitative approach that distinguished 8 different profiles based on prototypical motivations. Each profile was named using prototypic metaphors. In this case, the profile refers to the Charles Perrault classic fairy tale about a princess that was cursed to sleep waiting to be awakened by a handsome prince. In other words, we found in adolescents significantly more females whose main motivation for radicalization was the search of an ideal husband/love. They had a frequent history of abuse (22).

## Discussion

### Radicalized Minors and Radicalized Adults Have Different Profiles

The results of the comparison between radicalized minors and radicalized adults suggest that the 2 populations follow different paths while undergoing the radicalization process. These results seem to draw the lines of 2 distinct types of radicalization:

- *Among minors*: the subjects are mainly vulnerable adolescents who, through radical engagement *via* internet find a reassuring way to soothe their psychological suffering and their identity issues that are frequent during adolescence. This radical engagement probably could have been different if raised at another time or in another location, around other radical or extremist struggles (e.g. ETA, IRA, Hamas or The Red Brigade). In addition, the over-representation of females may be explained by the fact that there has been a specific recruitment of girls on the internet by jihadists and also by the fact that among young people, the profile of ‘Sleeping Beauty’ is more frequent (22): they are mostly young female single teenagers who are open for a relationship and are likely to be responsive to the recruiters’ inviting propositions.
- *Among young adults*: the radicalization process takes place in a physical way as a group phenomenon in specific neighborhoods. It appears to be more closely linked to the Islamism ideology and identity, social discrimination and marginalization (5). It may also be related to the questions of social polarization (23), the feeling of injustice and the perceived group threat (23). Radical ideologies rely on these societal phenomena to justify their violent actions. Of note, when we explored prognosis at follow-up with multivariate models in the same sample, we found that this last profile had the worst outcome (25).

### Comparison With Existing Literature

There have been several recent reviews and books trying to offer a global overview of the radicalization phenomenon (3, 5, 23, 24). To our knowledge, this study is the first to show quantitatively that there are some differences between radicalized minors and radicalized adults. Here we proposed to focus on the significant dimensions we found in the univariate comparisons: age, adolescence, gender, status at follow-up.

*Age effect*: Although age has been claimed as an important variable, studies with empirical data are missing. Bazex and Bénézech have underlined the age effect in the context of a study focusing on court petitions for radicalization (25). These authors showed an age effect on the reason of justice control. Subjects under judicial control for acts of apology or for acts of terrorism are much younger than those condemned for ordinary law crimes. On one hand, there are young people for whom radical engagement leads them to be under judicial control, and on the other side there are condemned adults who meet, by availability, radicalization in prison (25). This study corroborates our finding: for adults, radicalization seems to occur more likely through physical encounter such as imprisonment.

*Adolescence as a risk factor*: With a total population mean aged 19.82 years old, our study is in line with previous studies that highlighted the fact that youth is a risk factor for radicalization. Various authors explained how adolescence, *per se* – as a phase of turbulence and reorganization –, acts as a risk factor for radicalization (24). Because of the detachment from primary care givers and the necessity to find one’s own identity, adolescence can cause insecurity and sometimes a fear of loneliness and of being abandoned (22, 24). In response to this insecurity, belonging to a radical community conveys a sense of belonging, of meaning and comfort (19). Two similar proposals have been made to explain these adolescent trajectories. Based on the psychoanalysis of a radicalized subject and the content of jihadist propaganda, Leuzinger-Bohleber claims that the IS permits the satisfaction of pre-genital drives, which are rekindled in the early phases of adolescence (26). Violent actions advocated by radical groups unconsciously offer an enormous satisfaction of archaic drive-impulses and can be experienced as an omnipotent victory over the fear of death (26). The changes in identifications during adolescence and the quest for an ideal open the way to radical ideologies (24). Thus, the message sent by the IS may become attractive for some adolescents. The adolescent characteristics of the radicalization process had not been put forward in the terrorist movements of the 1990s and 2000s. Based on cognitive studies, other authors have claimed that personal uncertainty is one of the three main determinants of a radical belief system, along with perceived injustice and perceived group threat (23). This finding is based on Hogg’s ‘uncertainty-identity’ theory: the more individuals are uncertain in their environment, the more likely they are to identify themselves massively with groups (27) and the more the properties of this group form a unit where individuals seem interchangeable, the more effectively this group reduces uncertainty (28). Identity issues and personal uncertainty are psychological fragilities that are common during adolescence, especially when the young person lacks a structuring and supportive environment. This is why the group and its contextual and current ideology is an easy choice for a vulnerable teenager.

*Sex ratio*: The fact that there are more females among radicalized minors is confirmed by the existing literature. Finding love objects outside of the family is another major issue of adolescence and is simplified by the radical organization, which guarantees a reassuring marriage for females (22, 26). Motivation towards marriage is found more frequently in young girls than in young men who are generally more attracted by the fascination exerted on them by armed combat (16, 20, 29).

*Deradicalization*: In our study, there were less minors who were still considered as Muslim after deradicalization than adults (85.7 versus 97.5, p = 0.008). This result suggests that religious commitment is less stable in adolescents or that the deradicalization program has more profound effects in adolescents, although giving up one’s believe is not an aim of the deradicalization program. According to existing literature, it appears that identifications to radical group may be more labile for minors, as these identifications are closely linked to adolescent issues (19, 26). Therefore the deradicalization programs may be more efficient in this population, by offering the adolescent mind a way out of the radical commitment (30) and a response to his/her inherent vulnerabilities related or not to previous history (31).

### Limitations of the Study

As explained in the methods section, our sample included more females, adolescents, and convert Muslims than the current known distribution of radicalized individuals in France (25). Our study may include some selection bias. The initial request to the CPDSI was not done by judicial authorities but came from the subject’s environment (family, friends, neighborhood, school). The latter was more likely to perceive radicalization when the changes in habits or behaviors were obvious. This may contribute to the over-representation of women in our sample: a full veil is a clear visible element that may lead to the declaration to the CPDSI earlier than for male subjects with less detectable changes. Besides, we believe that in our sample, families showed more concern regarding these individuals as radicalism appeared to be dramatically opposed to their beliefs and background. This may imply an over-representation of families of cultural or religious origins different from Islam. Nevertheless, we had the same proportion of individuals who reached the IS (10%) as the national estimation (32). In addition, systematic declaration to the police may have altered the authenticity of some information despite the mixed method we used. We believe that keeping cases with only 5% of missing data facilitated analyzing the best informative cases.

## Conclusion

We concluded that radicalized minors appear to be more psychologically vulnerable individuals than radicalized adults. They are mainly vulnerable adolescents who try to deal with their identity issues and to soothe their distress through radical engagement. Thus, we believe that child and adolescent psychiatry has a role to play in countering violent extremism by (i) being involved in deradicalization programs; (ii) giving a meaning to radical engagement; and (iii) offering the adolescent mind a way out of the radical commitment. This should be done through a *tailored* and *multidisciplinary* intervention, collaborating with families, social services, justice and police. Society has a mission of child protection for minors and must do its best to protect its vulnerable citizens.

## Ethics Statement

The Center of Prevention against sectarian Drift related to Islam (CPDSI) was commissioned by the French government to support young people and their families who have been impacted by radicalization. This study was led by a specific Home Office decree directed to all heads of police departments (*Ministère de l’Intérieur*, *circulaire* INTA1512017J, 20 May 2015). Contact of the targeted population with the CPDSI was either direct or free through a national phone number (tel 800) or compulsory after contact with local police or under court petition. When individuals or families contacted the CPDSI freely, they were informed on all its services and responsibilities including compulsory declaration to the local police administration of all radicalized individuals. Consequently, some families and/or individuals were not inclined to share all requested information, and the dataset could not be fully informed immediately and for all individuals. Similarly, consent could not be obtained at first contact. However, for the research dataset, individuals (and their parents, in the case of minors) signed a consent form for the anonymous use of the data for research at some point during follow-up (FU).

## Author Contributions

NC, DB, DC, and GB designed the study. LB, DB, SH, and NC collected the dataset. LB, SH, and DB performed the qualitative analysis. DC, AO, and HP performed the quantitative analysis. NC, DB, GB, and AO made the literature search. AO, DC, NC, and GB drafted the MS. All authors reviewed and approved the MS.

## Funding

The study was funded by the French Home Office (*Ministère de l’Intérieur*, CIPD: Centre international de prévention de la délinquance) and the French Prime Minister Office (MIVILUDES: Mission interministérielle de vigilance et de lutte contre les dérives sectaires). NC was supported by a PhD grant from the groupe SOS and the Préfecture de Paris.

## Conflict of Interest Statement

LB and DB are currently employees of Bouzar-Expertises, a training firm dedicated to radicalization that offer training session for Justice, Police, Psychiatric, and Social Agency staffs on the topic of radicalization and radical Islamism.

The remaining authors declare that the research was conducted in the absence of any commercial or financial relationships that could be construed as a potential conflict of interest.
